# Genetic Studies on Multiple Consanguineous Families Segregating Diverse Phenotypes of Microphthalmia Identified Novel and Recurrent Mutations

**DOI:** 10.2174/0109298673365255250418092319

**Published:** 2025-05-05

**Authors:** Kashmala Samad, Fazeelat Samad, Abdulfatah M. Alayoubi, Aisha Siddiqua, Mohammed Turki Hussain Alharthi, Hussam Baghdadi, Sumra Wajid Abbasi, Muzammil Ahmad Khan, Muhammad Latif

**Affiliations:** 1 Gomal Center of Biochemistry and Biotechnology, Gomal University, Dera Ismail Khan, 29050, Pakistan;; 2 Department of Pathology, Gomal Medical College, Gomal University, Dera Ismail Khan, 29050, Pakistan;; 3 Department of Basic Medical Sciences, College of Medicine, Taibah University, Madinah, 42353, Saudi Arabia;; 4 Department of Biological Sciences, National University of Medical Sciences, Rawalpindi, Punjab, Pakistan;; 5 Center for Genetics and Inherited Diseases, Taibah University, Madinah, 42353, Saudi Arabia

**Keywords:** Microphthalmia, anterior segment dysgenesis, whole exome sequencing, *PXDN*, *FOXE3*, collagen IV

## Abstract

**Introduction:**

Anophthalmia/microphthalmia (A/M) and anterior segment dysgenesis (ASD) are severe ocular anomalies impacting eye morphology, occurring in 30 per 100,000 live births. Genetic research has identified over 30 genes linked to A/M anomalies, with their products mainly involved in eye organogenesis.

**Aims and Objectives:**

This study examined two consanguineous A/M families to identify disease-associated pathogenic mutations and predict their functional impact.

**Methodology:**

Patients were clinically examined using A-scan and ophthalmic ultrasonography. Whole exome sequencing (WES) identified candidate pathogenic variants validated through Sanger sequencing. Computational analyses assessed the impact of these mutations on protein structure and function.

**Results:**

The clinical diagnosis of family A revealed microphthalmia with ASD, while family B presented with an A/M phenotype. Exome analysis of family A identified a novel missense variant, NM_012293:c.A3742G [p.(Arg1248Gly)], in the peroxidasin (*PXDN*) gene (ClinVar ID: VCV001333267.1). At the cellular level, *PXDN* is involved in establishing sulfilimine bonds in collagen IV, a component of the basement membrane, suggesting that ocular defects may result from impaired integrity of the basement membrane in the developing eye. In contrast, Family B exhibited a nonsense variant NM _012186:c.720C>A (p.Cys240*) in the *FOXE3* gene. This variant has been previously reported in other South Asian populations, suggesting a founder effect in subcontinent populations. Structural modeling and simulation analysis of mutant proteins revealed altered properties, thus corroborating the pathogenicity of the identified mutation.

**Conclusion:**

Our findings may contribute to the elucidation of genotype-phenotype correlations, potentially facilitating the molecular diagnosis of microphthalmia and ASD.

## 1 INTRODUCTION

Anophthalmia/microphthalmia (A/M) is a rare ocular disorder that affects eye development [1]. Anophthalmia causes complete or partial absence of some or all parts of the eye tissue [2]. In contrast, microphthalmia is described as a hypoplastic or primitive condition with a corneal diameter <11 mm and an axial length of ~21 mm, respectively [3]. Based on the axial length, microphthalmos can be divided into three categories: mild (axial length ~17-21 mm), moderate (axial length ~12-16 mm), and severe (axial length <12 mm) [4]. Clinically, microphthalmia can be syndromic (multisymptomatic) or nonsyndromic (uni-symptomatic). In multisymptomatic conditions, A/M is generally associated with anterior segment dysgenesis (ASD), which is referred to as malformation of the cornea, lens, iris, or sclera. It is estimated that one-third to one-half of the reported A/M cases have syndromic microphthalmia [5, 6]. Isolated or non-syndromic microphthalmia may affect one eye (unilateral) or both eyes (bilateral) [7].

An estimated prevalence rate of 0.20 to 0.40 per 10,000 births has been recorded for anophthalmia, whereas microphthalmia has shown a prevalence range of 0.20 to 1.70 per 10,000 births [8]. Moreover, several epidemiological studies have shown that the combined prevalence rate of anophthalmia and microphthalmia is estimated to be 2.5 to 3.2 per 10,000 live births [9]. The etiology of microphthalmia may involve genetic and environmental factors [10]. Environmental factors may involve viral infections, such as varicella, rubella, and cytomegalovirus, the use of certain drugs and alcohol during gestation, or exposure to radiation [11]. Several genes linked to isolated or syndromic forms of microphthalmia have been identified. However, microphthalmia in ASD is rare and has a wide scope of investigation.

This study included two multiplex consanguineous Pakistani families, family A and family B, segregating A/M and ASD with variable intra-familial phenotypes. Family A exhibited a novel missense variant, NM_012293:c.A3742G, [p.(Arg1248Gly)], in the 18^th^ exon of the *PXDN* gene, while in family B, a homozygous nonsense variant NM 012186:c.720C>A (p.Cys240*) was identified in the *FOXE3* gene. This investigation may aid in elucidating possible genotype-phenotype correlation that will eventually assist in the molecular diagnosis of A/M and ASD.

## 2 MATERIALS AND METHODS

### 2.1 Ethical Approval and Family Selection

The ethical review board [DI-21-031, 10-05-2021/ (IRB# 04/ ERB/GU)] of Gomal University, Dera Ismail Khan, Pakistan, approved the current study, and written consent was obtained from the members of two enrolled families, family A and family B. These families were ascertained from different regions of D.I. Khan city in the Khyber Pakhtunkhwa (KP) province, Pakistan. Subsequently, the families’ pedigree was drawn based on the information collected from the respective parents. Later, the blood samples were collected, and DNA was isolated using a GeneJET Genomic DNA purification kit (Catalog # K0721, Thermo Fisher Scientific, USA) by following the manufacturer's protocols.

### 2.2 Clinical Diagnosis

For clinical-ophthalmic investigation, A-scan and B-scan ultrasounds were performed. The A-scan was conducted to determine the axial length and intraocular power of the lens, while the B-scan was utilized to examine the structural integrity of various ocular tissues, including retinal cells, vitreous, and optic nerve. Additionally, physical evaluation, vision assessment, and general ophthalmic examination were also performed.

### 2.3 Whole Exome Sequencing and Data Analysis

Whole exome sequencing (WES) was carried out to map the disease-associated mutation. The sequencing library was generated using the “xGen Exome Research Panel v2.0 Kit” (Integrated DNA Technologies, Coralville, Iowa, USA) and sequenced on a NovaSeq 6000 (Illumina, San Diego, CA, USA). FASTQ data files were converted to base calling files using bcl2fastq (v2.20.0.422). The BAM file was obtained by aligning the sequence reads with the reference genome (GRCh37/hg19) using BWA-mem 0.7.17 (arXiv: 1303.3997v2 [q-bio. GN]). Variant calling was performed using GATK (version 3.8) [12]. However, the annotation and interpretation of the variants were accomplished with the aid of the “evidence tool” (South Korea) [13]. wANNOVAR (https://wannovar.wglab. org/) [14] was used for variant annotation and Variant Studio software (v3.0). Several web-based tools were used to predict the biological significance of the candidate variants. These tools included MutationTaster [15], Polyphen2 [16], Provean [17], SIFT [18], and Panther [19].

### 2.4 Segregation Analysis

Validation of the identified disease variant through exome sequencing was performed *via* Sanger sequencing. For this purpose, primers were designed using the Primer3web tool (version 4.1.0) [20], and sequence analysis was performed using the BioEdit tool (version 7.0.5).

### 2.5 Protein Structure Modeling and Docking

A protein 3D structural model was constructed using the Alphafold3 tool [21], and Protein-protein interactions were performed using ClusPro [22]. The STRING database was used to determine protein interaction networks [23]. However, structural visualization was accomplished using Chimera 1.13.1 [24], PyMOL 2.3 [25], and LigPlot + (Version 2.1) [26].

### 2.6 Molecular Dynamics Simulations

Molecular dynamics (MD) simulations were carried out to predict the movement of atoms and molecules using the Amber Tool [27]. The stability of the wild-type and mutant variants was evaluated in conjunction with the docked complexes by measuring the Root Mean Square Deviation (RMSD) of the carbon atoms in the backbone, which measures the difference between the initial and final structural conformations of a protein’s backbone atoms and can be used to assess the stability of a protein in relation to its conformation.

Moreover, Root Mean Square Fluctuation (RMSF) was used to analyze the protein trajectories of both wild-type and mutant proteins in order to gain a better understanding of how mutation affects protein structure and function. The protein’s structural stability was determined by calculating the variations produced by the simulation, which increased with little changes.

## 3 RESULTS

The present ophthalmo-genetic investigation involved two multiplex consanguineous Pakistani families suffering from microphthalmia and ASD. Both of these families were of Saraiki origin and were ascertained from diverse locations of D.I. Khan City in the KP province of Pakistan. The family A pedigree comprised five generations, containing three consanguineous loops with nine affected individuals in consecutive 4^th^ and 5^th^ generations, respectively (Fig. **1**). Family B also consisted of five generations, harboring seven affected individuals in the 4^th^ and 5^th^ generations (Fig. **2a**).

### 3.1 Clinical Findings

#### 3.1.1 Family A

The patient exhibited bilateral microphthalmia, staphyloma, sclerocornea, and ASD. However, minor intra-familial phenotypic variability was observed among the patients. During the early childhood of patients, superficial vision was present, which was reduced with the passage of time, and vision loss ultimately occurred. The lens was deformed in shape, indicating a moderate cataract phenotype. The retinal examination was not possible because of corneal opacity. Nevertheless, the parents of all consanguineous loops were asymptomatic with no medical history of any eye anomaly. The B-scan of the patients showed no retinal detachment, no tumor growth, and a clear vitreous without hemorrhagic lesions. Fig. (**1b**) shows the clinical images of the affected individuals of family A. The clinical findings for each patient are summarized in Table **1**.

#### 3.1.2 Family B

In the case of family B, some of the affected individuals had anterior staphyloma and sclerocornea, while others exhibited only microphthalmia. Visually, the patients were blind and had poor light perception (Fig. **2b**). Likewise, the B-scan of the patients belonging to family B revealed no retinal detachment, no tumor growth, and a clear vitreous without hemorrhagic lesions (Fig. **2c**). However, the patient IV-10 of family B had unilateral microphthalmia of the left eye with a mass of 0.8 cm x 0.6 cm measurement seen within the vitreous. Similarly, the A-scan examination of patient IV-10 found an 18.15 mm axial length of the eye globe. Similarly, the B-scan findings in patient V-10 did not reveal retinal detachment or tumor growth. The vitreous was clear without any hemorrhagic lesions. This patient also had unilateral microphthalmia of the left eye with a mass of 0.9 cm x 0.5 cm measurement. However, no flow was observed in the mass. Furthermore, the A-scan demonstrated an axial length of 15.94 mm. In patients with staphyloma and sclerocornea, examination of the optic disc, lens, and iris was not possible (Fig. **2c**). Despite these findings, intrafamilial phenotypic variability was observed among different affected individuals. Table **2** describes the diagnostic findings of the affected individuals used to elucidate the phenotypic spectrum.

### 3.2 Molecular Findings

#### 3.2.1 Family A

Exome sequence analysis of this family has proven to be a valuable method for diagnosing genetic disorders by identifying the genetic mutations that cause diseases in individuals. This enables an accurate diagnosis and plausible management. Whole exome data analysis revealed a homozygous missense variant, NM_012293:c.A3742G [p.(Arg1248Gly)], in the 18^th^ exon of *PXDN (ClinVar ID: VCV001333267.1)*. Sanger sequencing of additional family members (including parents) confirmed their segregation with the disease phenotype (Fig. **1c**). The missense variant substituted the highly conserved arginine at position 1248 with glycine. Hence, multiple protein sequence alignments confirmed its evolutionary importance across animal species.

#### 3.2.2 Family B

Whole exome data analysis of this family revealed a homozygous nonsense variant NM_012186 c.720C>A, (p.Cys240*) in the *FOXE3* gene. Sanger sequencing of patients and normal individuals (Fig. **2d**) confirmed its segregation with the disease. The reported variant presumably removed a C terminal peptide downstream to the forkhead domain.

### 3.3 Structural and Interactional Findings of Normal and Mutant Proteins

#### 3.3.1 Family A

The predicted 3D structures of wild-type and mutant *PXDN* failed to superimpose with each other and showed a similarity index of 0.2%. This suggests the loss of its native tertiary conformation (Fig. **3**). Protein interaction studies on *PXDN* (both normal and mutant) and its closely related MYT1L protein revealed altered docking sites with respect to residue number, residue identity, and bonding type. The normal *PXDN* protein interacts with the MYT1L protein through 26 amino acid residues (Glu900, ASP37, Pro34, Glu97, Glu147, Gly35, Glu1068, Glu1081, Asp1067, Asp1074, Asn1077, His1023, Lys1050, Lu840, Thr1033, Lys1040, Gln1051, Glu1061, Glu144, Asp145, Ala 1053, Glu1058, Asn 1055, Asp1060, Ser1054, and Arg105) through 37 H-bonds and three salt bridges. In contrast, it was found that the mutant *PXDN* protein interacted with MYT1L through 22 amino acid residues (Arg283, Gln292, Ser 230, Asp293, Asp151, Glu155, Asn284, Asn281, Gln 270, Glu159, Asp65, Glu284, Gln64, Glu166, Glu140, Glu70, lu164, Glu165, Asp143, Glu144, Glu163, and Glu162) *via* 32 H-bonds and seven salt bridges (Fig. **3**).

#### 3.3.2 Family B

The predicted 3D structures of wild-type and mutant *FOXE3* failed to superimpose with each other, indicating the loss of its normal conformation due to the protein truncating mutation (Fig. **4**). The percent similarity index was found to be 44.7%, indicating that the mutant protein has undergone significant alterations from the wild-type protein. Protein-protein interaction studies of normal protein and mutant *FOXE3* protein with its close interactor PITX3 protein revealed significant changes in the docking sites. Compared to the wild-type protein, the mutant *FOXE3* protein showed a unique interaction pattern in relation to residue number, residue identity, and bonding type. The normal protein interacts with PITX3 protein through 14 amino acid residues (Arg166, Ser216, Leu216, Arg162, Arg163, Arg113, Leu164, Lys71, Lys114, Arg62, Gly60, Arg61, Pro57, and Glu50) and 23 H-bonds. In contrast, it was found that mutant protein interacted with PITX3 through 10 amino acid residues (Lys114, Pro187, Arg162, Ala162, Ala23, Ser75, Ala230, Arg120, Arg166, and Lys165) and 12 bonds, that is, 10 H-bonds and two salt bridges.

### 3.4 Molecular Dynamic Simulations

#### 3.4.1 Family A

To check the stability of both wild-type and mutant systems, the RMSD value for the carbon-α atoms was computed during a 25 ns simulation (Figs. **5a**, **b**). The wild-type system displayed unstable behavior with high and low fluctuations from the start of the simulation. These fluctuations were observed until the end of the 25^th^ ns simulation, with an average RMSD of 11.63 Å. In the case of mutant protein, equilibration was attained at 11.9 ns, and the system remained stable with an average RMSD value of 10.25 Å till the end of the simulation. The trend showed in the RMSD graphs of both systems revealed that the mutant *PXDN* exhibited stability over a period of 25 ns, whereas the wild-type fluctuated significantly. Furthermore, RMSF was used to identify the regions of proteins with high levels of flexibility. The residue-based root means square deviation (RMSF) of the backbone for the wildtype system displayed more flexible residues with an average value of 4.61 Å as compared to the mutant *PXDN* system (4.73 Å). In the wild-type protein, the peak at residue 1248 was more flexible, with a mean RMSF value of 3.34 Å. However, in the mutant protein, the same peak was more defined, with an average value of 2.26 Å, indicating reduced flexibility (Fig. **4**). Molecular dynamic simulation analysis also validated the genetic findings of novel missense mutations in *PXND* that cause microphthalmia and ASD.

#### 3.4.2 Family B

As illustrated in Fig. (**5**), both examined systems exhibited varying degrees of fluctuation at first but ultimately achieved equilibrium and demonstrated stable behavior until the end of the MD simulation. Both curves were observed to be stable with initial fluctuations, and the average RMSD value recorded for the wild-type was 4.5 Å, and the mutant was 3.5 Å. To assess the residual fluctuation, an RMSF graph was obtained for the wild type, giving its peak value of 8.6 Å, while the mutant did not show any change. ROG (radius of gyration) graph depicting the compactness and relaxation of the structure was obtained for wild-type and mutant ones. The maximum peak of wild-type was observed at 4.9 Å at 8 ns and remained stable between 20-40 ns, and then changes were observed at 50 ns. RoG cure for the mutant remained stable throughout the simulation with 3.5 Å of the average value (Figs. **5c**, **d**).

## 4 DISCUSSION

### 4.1 Functional Description of *Pxdn* and its Disease Association

*Pxdn* encodes 1479 amino acids long “peroxidasin protein”. It is expressed in the corneal endothelium during the embryonic stage, whereas during the adult stage, it is expressed in the corneal and lens epithelia [28]. Genetic variations in the *Pxdn* gene lead to different congenital eye disorders, such as cataracts, corneal opacification, anophthalmia, developmental glaucoma, and ASD. Anterior segment abnormalities may involve iris hypoplasia, corneal vascularization and opacity, polycoria, abnormal iridocorneal angle, ectopia lentis, corectopia, and several developmental anomalies of the eye tissue. These symptoms may appear either alone or in combination with other symptoms. Some clinical conditions like axenfeld anomaly [29], aniridia, Rieger syndrome [30], and iridogonio-dysgenesis [31, 32] fall into anterior segment dysgeneses. *Pxdn* gene mutations have also been found to be involved in certain cancers, such as metastatic brain cancers, melanoma breast cancer, ovarian cancer, colon cancer, and renal cancer [33-35].

In a study [36], two *Pxdn* knockout models were constructed to investigate the effect of *Pxdn* mutations on eye development and disease. The first *Pxdn* model was generated by removing the first exon using the CRISPR technology. In homozygous mice, loss of *Pxdn* expression and collagen IV sulfilimines were observed, which eventually led to entirely closed eyes, extremely reduced vision, and external morphological disorders in other parts of the body. Histological examination revealed severe eye defects, including the absence of eyeballs or an extremely aborted eye structure in homozygous mice. However, heterozygous mice displayed a normal eye structure. The visual acuity of the heterozygous mice did not show any apparent functional impairment. These results suggest that *Pxdn* activity has an important functional impact on eye development [36]. The second *Pxdn* model was created to explore the function of the LRR domains during eye development. In this model, two exons encoding the LRR domains were deleted. In this knockout model, deletion of the LRR3 and LRR4 domains caused severe microphthalmia, ASD, cataracts, and progressive glaucoma [36, 37].

Peroxidasin plays a major role in hydrogen peroxidase metabolism. Peroxidasin catalyzes the synthesis of a sulfilimine bond between the apposed Lys and Met amino acids in the C-terminal domain of collagen IV, which is the main part of the basement membrane [38]. It has been reported that defects in peroxidasin impart defective basement membrane reliability in *Caenorhabditis elegans* and fruit flies. Mutations in human *PXDN* are also expected to affect the integrity of the basement membrane of the lens and cornea by unsettling the development of collagen IV. Genetic haploinsufficiency of collagen IV has been shown to cause variable clinical features of ASD in mice and humans. Variable clinical phenotypes may include cataracts, corneal opacities, iris defects, iridocorneal adhesions, and buphthalmos, similar to those observed in patients with *PXDN* gene defects [39-41].

To date, 49 diverse mutations have been identified in the *PXDN* gene, comprising 22 missense mutations, three splice sites, seven small deletions, four small insertions, and one small deletion mutation (HGMD Professional 2023.4). Clinically, these mutations are associated with microphthalmia, ASD, cataracts, glaucoma, and corneal opacity (Human Gene Mutation Database, accessed in March, 2023). In the current study, we report a novel missense variant, NM_012293:c.A3742G [p.(Arg1248Gly)], in the 18^th^ exon of the *PXDN* gene. The recruited patients were presented with intrafamilial variable phenotypes of congenital microphthalmia, sclerocornea, and staphyloma with ASD. However, patients with *PXDN* gene mutations have also been shown to exhibit ASD, sclerocornea, microphthalmia, hypotonia, and developmental delays [8, 32]. The current study also reported that *PXDN* gene mutation is involved in the development of variable clinical features of microphthalmia and ASD. These findings are consistent with those reported in studies on mouse models and previously characterized patients with segregating *PXDN* gene mutations [32, 39]. Since microphthalmia with ASD is a rare condition with a few identified genetic factors, extensive screening is required to expand the genetics of this condition to explore genotype-phenotype associations.

### 4.2 Functional Description of *FOXE3* and its Disease Association

*FOXE3* is a “fork-head box E3 protein”, which produces a single transcript that consists of a single exon, which encodes a 319 amino acids long protein [42, 43]. Studies on zebrafish and mice have shown that *Foxe3* is expressed during the developmental stage of the ocular lens. In mice, *Foxe3* is expressed in the early stage of the lens placode, and later, it is confined to the anterior epithelium of the lens. Additionally, *Foxe3* is also shown to be expressed in the forebrain, midbrain, and pharyngeal arches during mouse development [44, 45]. Various mouse models exhibiting either a gain or loss of functional activity have been described. These include transgenic mice with *Foxe3* expression in the lens determined by Cyraa, promoter in lens fiber cells, and mice with spontaneous mutations, such as dyl and rct (Rinshoken cataract), as well as two distinct *Foxe3*-targeted gene knockout mouse models [46-48]. Mouse mutants with heterozygous dyl mutants were found to have some lens and corneal defects, along with symptoms of Peter’s anomaly. Mutant mice with homozygous rct showed reduced expression of the *Foxe3* gene, which led to cataracts and microphthalmia. Gene expression analyses of the *Foxe3* null mouse model revealed that *Foxe3* is necessary to promote cell proliferation by inhibiting their proliferation into fiber cells. Ablation of *Foxe3* causes impaired fiber development and activates apoptosis, resulting in aberrant spaces in the posterior region of the lens [45]. In zebrafish, *Foxe3* knockdown results in reduced lens size characterized by many layers of epithelial cells and aberrant fiber cells. In contrast, *Foxe3* mutant mice and zebrafish antisense morphants exhibit defects in epithelial cell proliferation, with an accumulation of undifferentiated epithelial cells. Moreover, studies on mice have also reported that Pitx3 has a significant role in regulating the *Foxe3* gene expression during early lens development [49, 50].

In the recent two decades, 52 studies have identified 33 different mutations in *Foxe3*. These mutations are classified as frameshift (06), missense (23), and stop-gain (04) mutations. Of these reported mutations, 15 missense variants were present within the forkhead domain (Human Genome Mutation Database: HGMD.cf.ac.uk/ac/all.php). A variety of eye phenotypes, like aphakia, sclerocornea, anterior segment dysgenesis, and microphthalmia, have been linked with dominant or recessive mutations in the *FOXE3* gene [51, 52]. Earlier, seven different mutations have been described in *FOXE3* to be involved in different ocular phenotypes in Pakistani patients. In a molecular study, a patient suffering from aphakia was found to have a premature nonsense mutation [p.(Cys240X)] in the *FOXE3* gene [53]. Likewise, three further Pakistani families inheriting bilateral anophthalmia reported homozygous mutation [p.(Met7Ilefs*216)] and p.Ile97Val) in *FOXE3* [54]. Furthermore, two homozygous missense mutations [p.(Asn117Lys) and p.(Glu103Lys)] in *FOXE3* involved in Peters anomaly were also identified in Pakistani families [55]. Another genetic study on a Pakistani family found the same homozygous missense variant (p.(Glu103Lys)) in *FOXE3* to be involved in autosomal recessive congenital cataracts [56]. Recently, a homozygous missense variant (p.(Ile97Val)) in *FOXE3* underlying congenital anterior segment dysgenesis, keratoconus, congenital bilateral corneal haze, and apparent microphthalmia in five affected individuals belonging to a large consanguineous Pakistani family has been reported [57, 58]. Akbar *et al.* (2024) recently mapped a Pakistani family with six affected persons showing multiple ophthalmic features (such as anophthalmia associated with other sensory processing disorders, including sensorineural hearing loss, aphasia, developmental delay, behavioral issue, and microcephaly). Mutation mapping in this family found a novel nonsense p.Glu36* variant in *FOXE3* and a missense variant in *AP4M1* [58]. The identified mutation p.Glu36* are predicted to be located upstream to nuclear localization signal and the forkhead domain. Protein structure analysis predicted that the reported mutation may alter the protein structure and lead to loss of function. Reis *et al.* (2021) performed protein expression analysis on p.Cys240*, p.Phe186fs, and p.Glu236fs using Western blotting and found that these protein-truncating mutations are responsible for reduced expression of mutant protein compared to wild-type [59].

A previously known missense variant c.720C>A [p.(C240X)] in the *FOXE3* gene was identified in the family B presented in this study. The recruited patients were presented with intrafamilial variable phenotypes of congenital microphthalmia, sclerocornea, and staphyloma. However, patients with *FOXE3* mutations have also been shown to exhibit aphakia, dysplastic irides, glaucoma, and sclerocornea. Our findings suggest that *FOXE3* mutations are a significant contributor to microphthalmia, with additional variable phenotypes. The *FOXE3* gene mutation that causes microphthalmia primarily exhibits an autosomal recessive pattern of inheritance [60]. The inter- and intra-familial phenotypic variability might be due to distinct familial backgrounds, patient’s age, nature and position of the variants, and/or additional allelic effect of other unknown interacting genes. Nevertheless, genotype-phenotype analysis revealed that protein-truncating mutations in the *FOXE3* gene are mostly associated with microphthalmia, staphyloma, and sclerocornea.

## CONCLUSION

In this study, a novel missense mutation, NM_012293:c.A3742G [p.(Arg1248Gly)], in *PXDN* and a recurrent nonsense variant, NM_012186:c.720C>A; p.(Cys240X)], in the *FOXE3* gene were found to be associated with bilateral microphthalmia and ASD. Protein structural modeling and molecular dynamic simulation analysis of identified mutations clearly indicated the altered pattern of mutant protein compared to normal, which explains that the identified mutation may lead to the disease phenotype. In the current study, the 5^th^ novel mutation of *PXDN* in a consanguineous Pakistani family was found. The current finding may help to establish a genetic diagnostic test for families affected by microphthalmia. Further clinico-genetic investigations are required to understand the variable intra-familial phenotypes caused by *PXDN* or *FOXE3* gene mutations. Moreover, the current study also signifies the diagnostic efficacy of whole exome sequencing in delineating the exact ophthalmic condition where definite clinical diagnosis is difficult.

## AUTHORS’ CONTRIBUTIONS

The authors confirm their contribution to the paper as follows: study conception and design: MAK; data collection: AMA, MTHA, and FS; analysis and interpretation of results: KS and SWA; draft manuscript: AS, HB, and ML. All authors reviewed the results and approved the final version of the manuscript.

## Figures and Tables

**Fig. (1):**
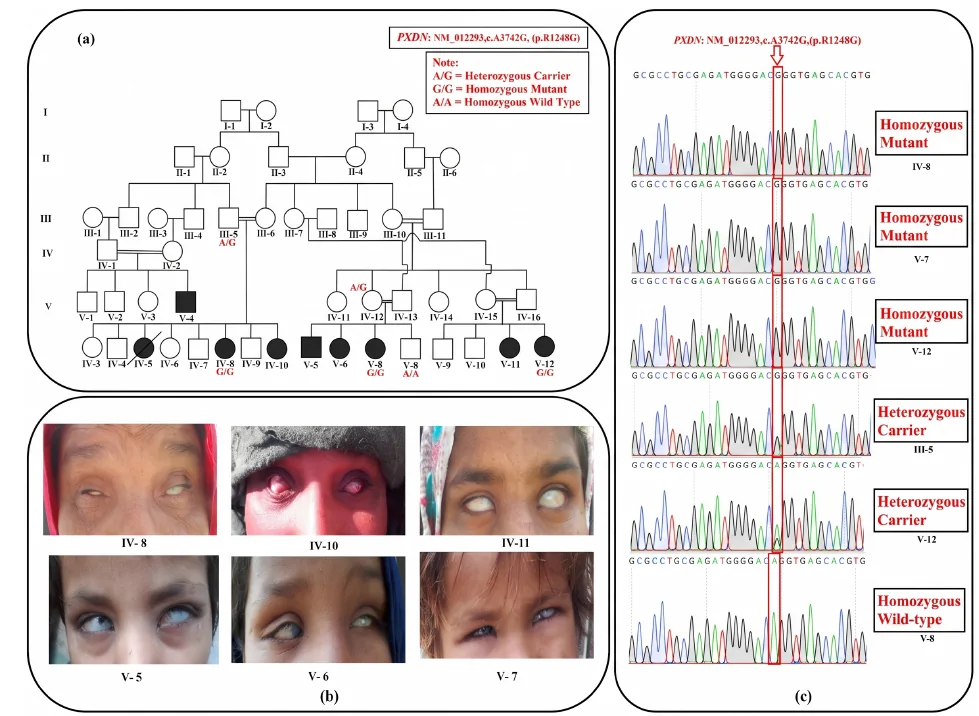
(**a**) The image depicts the pedigree of a five generation consanguineous family with multiple affected individuals. (**b**) Photograph of patients presenting bilateral microphthalmia phenotype. (**c**) Sequence chromatograms of identified mutation.

**Fig. (2):**
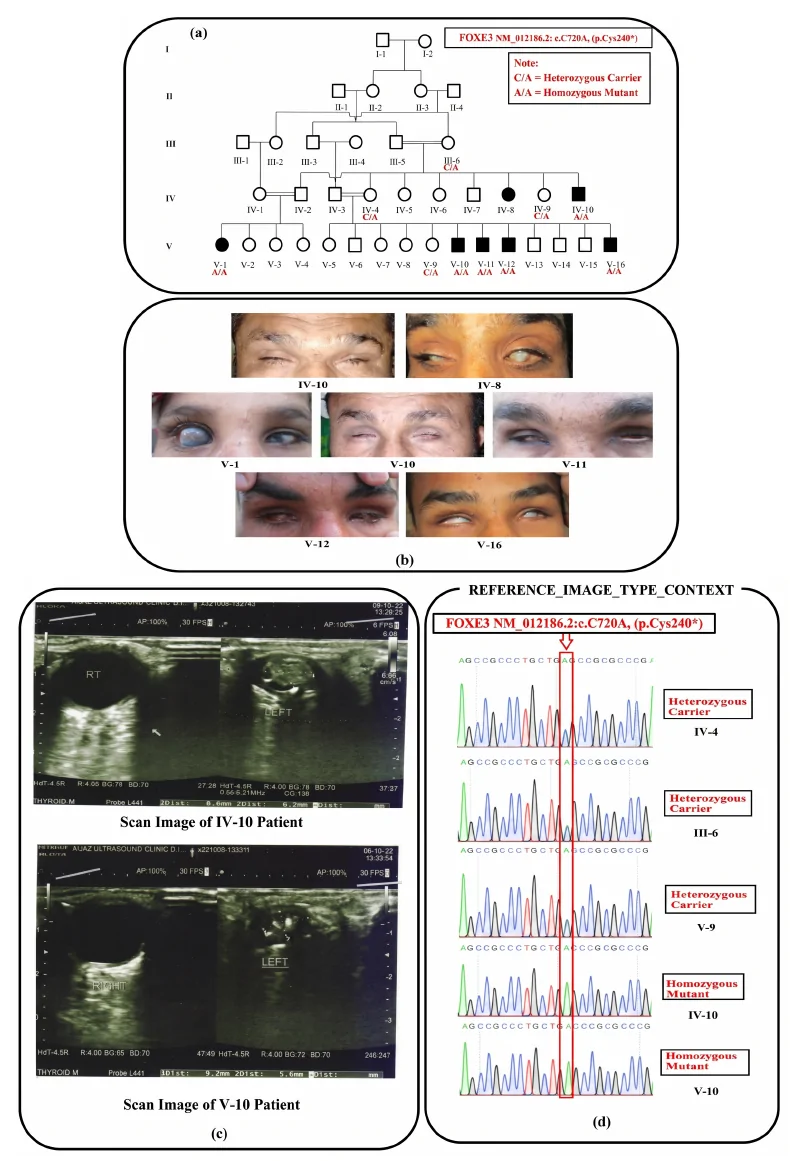
(**a**) Pedigree analysis illustrates an autosomal recessive mode of disease segregation, and the genotype status of each analyzed individual is represented as A/A (homozygous substitution) and A/C (heterozygous carrier). (**b**) Patients’ photographs and (**c**) B-scan images of patients IV-10 and V-10. (**d**) Sanger sequencing results show segregation of mutation in the family.

**Fig. (3):**
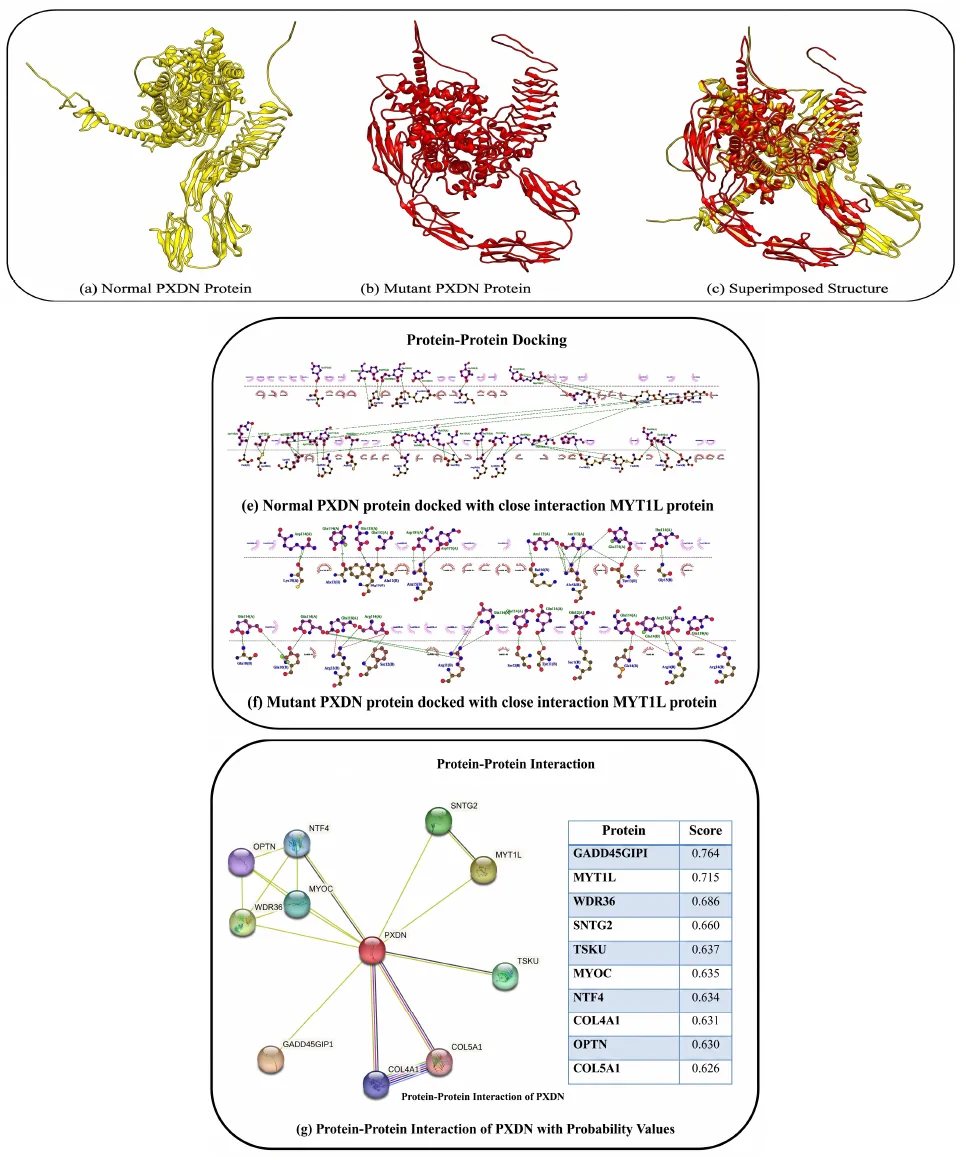
(**a**) Normal protein model, (**b**) Mutant protein model, (**c**) Superimposed models of wild type and mutant. (**d**) The lower panel shows wild-type and (**e**) mutant proteins docked with close interactor protein. (**f**) The left panel shows an image of STRING protein interaction depicting the close interactor of *PXDN* protein.

**Fig. (4):**
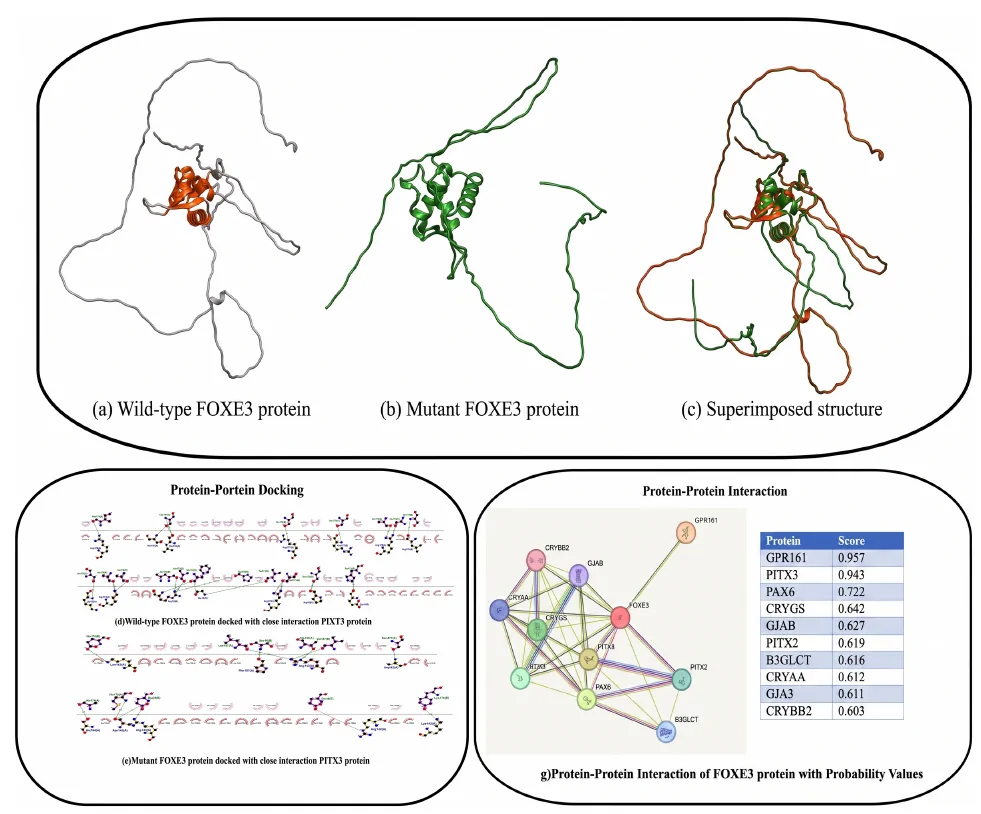
(**a**) Normal protein model, (**b**) Mutant protein model, (**c**) Superimposed protein model. (**d**) the lower left panel shows a docking image of normal and (**e**) mutant protein docked with a close interactor. (**f**) The lower right panel of the image shows a close interaction of FOXE3 protein.

**Fig. (5):**
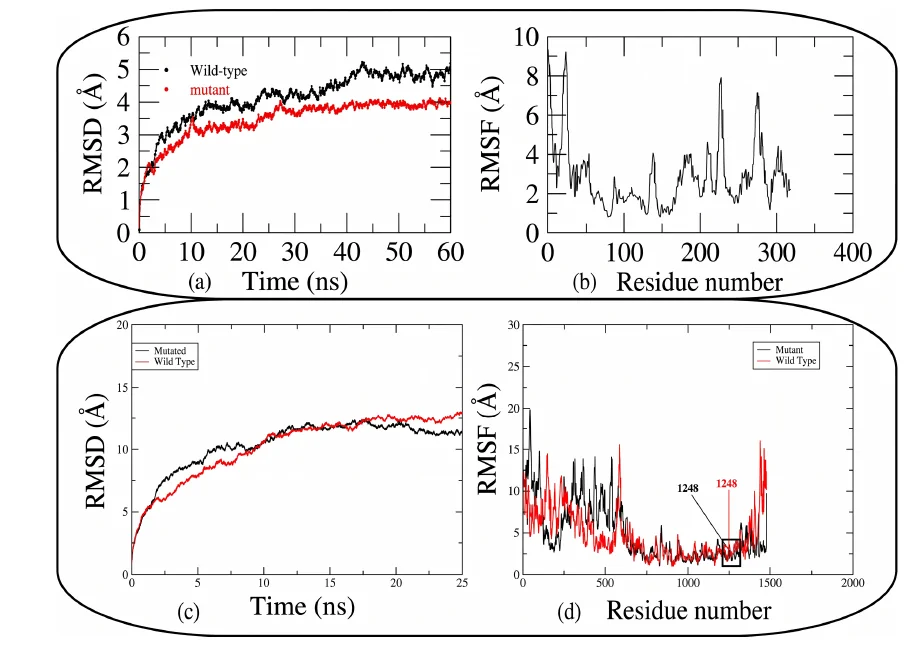
(**a-d**) The graph shows root mean square deviation and fluctuation to indicate the altered simulation status of wildtype and mutant *PXDN* (upper panel) and *FOXE3* protein (lower panel).

**Table 1:** Clinical findings of affected individuals under investigation in family A.

**Patient ID**	**Clinical Findings**
IV-8	(i)-Bilateral microphthalmia, (ii)-Corneal opacification, (iii)-Right eye was more affected than the center, (iv)- Lens dysmorphology
IV-10	(i)-Corneal opacification, (ii)-Staphyloma
IV-11	(i)-Sclerocornea, (ii)-Corneal opacification, (iii)-Strabismus, (iv)- Staphyloma
V-5	(i)-Strabismus, (ii)-Bilateral microphthalmia
V-6	(i)-Strabismus, (ii)-Bilateral microphthalmia, (iii)- Lens dysmorphology
V-7	(i)-Strabismus, (ii)-Bilateral microphthalmia

**Table 2:** Diagnostic findings of A-scan and B-scan of affected individuals IV-10 and V-10 in family B.

**Pathological Findings**	**Patient IDs**	
	**IV-10**	**V-10**
Axial Length	18.5 mm	15.94 mm
Pdol	35.5-06	43.00-06
Retinal Detachment	Not Seen	Not Seen
Abnormal Growth	Not Seen	Not Seen
Vitreous	Clear	Clear
Vitreous Hemorrhage	Not Seen	Not Seen
Mass	0.8 cm x 0.6 cm	0.9 cm x 0.5 cm
Flow	Not Seen	Not Seen
Fluid	Not Seen	Not Seen
Tumor	Not Seen	Not Seen

## Data Availability

Raw data is available upon request. Data associated with this study was not deposited into a publicly available repository. Reference sequences were obtained from freely available genome databases.

## LIST OF ABBREVIATIONS

WES: Whole Exome Sequencing
ASD: Anterior Segment Dysgenesis
MD: Molecular Dynamics
RMSD: Root Mean Square Deviation
RMSF: Root Mean Square Fluctuation
